# Augmented rescue of macroglobulins by supplementation of anti-snake venom with methanolic extract of *Andrographis paniculata* in *Naja naja* envenomation

**DOI:** 10.1007/s13205-022-03379-w

**Published:** 2022-10-05

**Authors:** Akshatha G. Nayak, P. Ashwini Aithal, Nitesh Kumar, Smita Shenoy, Maya Roche

**Affiliations:** 1 Division of Biochemistry, Department of Basic Medical Sciences, Manipal Academy of Higher Education, Manipal, Karnataka 576104 India; 2 Division of Anatomy, Department of Basic Medical Sciences, Manipal Academy of Higher Education, Manipal, Karnataka India; 3 Department of Pharmacology and Toxicology, National Institute of Pharmaceutical Education and Research (NIPER), Hajipur, Bihar India; 4 Department of Pharmacology, Kasturba Medical College, Manipal Academy of Higher Education, Manipal, Karnataka India

**Keywords:** Alpha 2-macroglobulin, *Andrographis paniculata*, *Naja naja*, Supplementary action, Polyvalent anti-snake venom

## Abstract

Proteins of the macroglobulin family are prime targets of venom enzymes in snake bite. A massive reduction in the active concentration of these multifunctional proteins in snake bite, makes the living system vulnerable to dysregulation. This study investigates the ability of Indian polyvalent anti-snake venom (ASV), methanolic extract of *Andrographis paniculata* (MAP) and their combination in rescuing human alpha 2-macroglobulin (A2MG) and its homologues in rat plasma, from inactivation by *Naja naja* (N.N) venom enzymes. In-vitro experiments were conducted with heparinized human plasma and in-vivo experiments with female Wistar rats. Along with appropriate controls, there were 3 test groups in in-vitro and 8 test groups in in-vivo experiments. The in-vitro test groups were exposed to N.N venom for zero, 30 or 90 min prior to incubation with ASV or MAP or reduced ASV supplemented with MAP and incubated for 16 h at 37 °C. Chymotrypsin-bound esterase (CTBE) activity of A2MG was estimated. Rats were administered the venom intramuscularly and treated with ASV/MAP/ASV + MAP. CTBE activity of macroglobulin homologues was measured on day 1, 7 and 14. Survival of animals was noted. In human plasma, addition of ASV or MAP or ASV + MAP prevented loss of A2MG activity maximally to the extent of 88–100% (*p* = 0.001). In rats, reduced concentration of ASV supplemented with MAP showed complete rescue of macroglobulin homologues and 90% survival. The compulsive evidence from this study, underscores the merits of using this multipronged strategy in rescuing the macroglobulins and improving survival in envenomation due to N.N.

## Introduction

Among the proteins that have evolved in nature with the principal motive of defending the intra and extracellular compartments of the living system against a proteinase onslaught, macroglobulins and their homologues are some of the most prominent. Macroglobulin homologues have been highly conserved in nature and found in most animal species (Rehman et al. 2013). In the rat, macroglobulin homologues exist in 3 different forms viz. alpha 2-macroglobulin (A2MG, a tetramer with molecular weight 720 KDa), is a positive acute phase reactant (Ito and Kuribayashi 2019), alpha 1-macroglobulin (a tetramer with molecular weight 720 KDa), is a non-acute phase reactant and alpha-1 inhibitor 3 (α1I3, a monomer with molecular weight 190 KDa), is a negative acute phase reactant (Lonberg-Holm et al. 1987; Enghild et al. 1989; Rehman et al. 2013). In humans, the macroglobulin family is represented by a single protein which exists as A2MG (a tetramer with molecular weight 720 KDa) (Feldman et al. 1985; Rehman et al. 2013). All of them have a striking unifying feature of having a thiol-ester site (Lonberg-Holm et al. 1987) which is susceptible to cleavage by a wide array of proteinases leading to formation of enzyme-inhibitor complex. A2MG works like the ‘Venus fly trap’ and has been shown to be involved in the regulation of multiple endogenous processes such as blood coagulation, fibrinolysis (Rehman et al. 2013), complement activation (Naseraldeen et al. 2021), antigen presentation (Binder et al. 2001) and inflammation (Vandooren and Itoh 2021). It is also known to regulate activities of cytokines, growth factors (Nancey et al. 2008; Peslova et al. 2009; Solchaga et al. 2012) and hormones (Birkenmeier et al. 1998; Fisker 2006; Peslova et al. 2009). A2MG is the first line of defense against proteinases arising from endogenous cell turnover as well as those which enter the body by way of microorganisms or venomous bites. Any damage to the living system by enzymes can occur only when A2MG is surmounted by an extremely powerful enzymatic onslaught or by action of enzymes which have no affinity for this protein (Kamiguti et al. 1994) as seen in venomous snake bites. Snake venoms are essentially rich cocktails of multiple enzymes, with varied catalytic activities. Being a pan-proteinase inhibitor, A2MG can trap proteinases belonging to all four classes viz., serine, cysteine, metallo and aspartic (Rehman et al. 2013). Hence, it is but natural that A2MG acts as a strong defense barrier in envenomation. In-vitro studies with crude venoms have demonstrated human A2MG to be a prime target of elapid venom enzymes from *Naja naja* (N.N) (Sujatha et al. 1988), *Naja nigricollis* (Evans and Guthrie 1984) and *Ophiophagus hannah* (King cobra) (Roche and Pattabiraman 1990). Purified venom enzymes such as, hemorrhagic metalloproteinase HR1 from *Protobothrops flavoviridis* (Morine et al. 2008), pictobin from *Bothrops pictus* (Vivas-Ruiz et al. 2020), *Bothrops asper* metalloproteinase BaP1 (Escalante et al. 2004) to name a few, are all inactivated by A2MG. After binding, the A2MG-enzyme complex is taken to the liver and destroyed. Hence any treatment strategy that would support the rescue of A2MG and its homologues and prevent dangerously low concentrations of this protein in biologic fluids, would have a better outcome in envenomation, by sparing this protein for its normal function as a ‘Physiological guardian’ (Rehman et al. 2013).

The standard treatment for envenomation with *Naja naja* (N.N) in India, is a polyvalent anti-snake venom (ASV). This polyvalent ASV is active against the venoms of the ‘Big four’ snakes of India, such as *Naja naja* (N.N, Indian spectacled cobra), *Daboia russelii* (Russell's viper), *Echis carinatus* (saw scaled viper) and *Bungarus caeruleus* (Indian krait) (Pore et al. 2015). Monovalent ASV against N.N is not manufactured in India. The use of the Indian ASV is associated with allergic and anaphylactic reactions in 20–50% (Deshpande et al. 2013; de Silva et al. 2016) and 10–15% (Deshpande et al. 2013) of envenomed patients respectively. Issues with logistics of transportation and storage affect the availability of ASV in rural areas, where most of the bites occur (Deshpande et al. 2013; Peshin 2012). In addition, the ASV is expensive and the cost of treatment along with hospitalization is considerable (Peshin 2012).

The effect of plant extracts in inhibiting snake venom enzymes is well documented (Carvalho et al. 2013; Dar et al. 2016; Coriolano de Oliveira et al. 2016; Saavedra et al. 2018; Amos Samkumar et al. 2019; Nayak et al. 2020). Compounds such as diterpene lactones, flavonoids, steroids, tannins and polyphenols found in plant extracts have been demonstrated to bind to venom enzymes (Carvalho et al. 2013; Dar et al. 2016), inhibiting them, as exemplified by docking studies (Sivakumar and Manikandan 2015; Amos Samkumar et al. 2019). *Andrographis paniculata* (A.P) or ‘King of bitters’ is one such medicinal plant, with numerous applications in different systems of medicine all over South Asia (M 2013; Gopi et al. 2014; Okhuarobo et al. 2014; Gnaniah 2014). Its use in the treatment of N.N bite is well documented. Among the various extracts of A.P, the methanolic extract (MAP) showed best survival time in envenomed mice (Gopi et al. 2011). In-vitro studies using MAP have shown neutralizing properties against various N.N venom enzymes such as acetylcholinesterase, hyaluronidase, phosphodiesterase, phosphomonoesterase, phospholipase A_2_ and L-amino acid oxidase (Sivakumar and Manikandan 2015). In-vivo experiments using MAP have shown anti-venom properties by reducing edema (Gnaniah 2014) and hemorrhage in mice (Meenatchisundaram 2009). Ethanolic extract of A.P when fed orally, prolonged survival time in Swiss albino mice (Premendran et al. 2011). Recent work using thromboelastography of human plasma demonstrated the efficacy of MAP in normalizing hemostasis and fibrinolysis in the presence of N.N venom (Nayak et al. 2020a). The extract was found to be very efficient in supplementing the action of ASV in normalizing prothrombin time (measure of extrinsic pathway of clotting), activated partial thromboplastin time (measure of intrinsic pathway of clotting) and also in inhibiting the catalytic activity of venom hyaluronidase (spreading factor) and acetylcholinesterase (AChE, a component of neurotoxicity) (Nayak et al. 2020, 2021). Hence it was thought worthwhile to investigate the effect of supplementation of ASV with MAP on human A2MG activity in the presence of N.N venom in-vitro. The study also investigates the effect of supplementation of ASV with MAP on the activities of A2MG and its homologues in-vivo in envenomed rats.

## Materials and methods

### In-vitro studies

#### Blood samples

After obtaining institutional ethics committee approval (IEC-320/2017) and written consent from healthy human volunteers (*n* = 3), blood samples were collected in heparinized vacutainers. Blood was then centrifuged at 2000 rpm for 10 min, plasma was separated and used for the study.

#### *Naja naja* venom

Lyophilized N.N venom was procured from KV Institute Ballia, Uttar Pradesh, India. Venom stock solution was prepared by dissolving 10 mg of the venom in 1 ml 0.08 M phosphate buffer, pH-7.6 and stored at 2–8 °C. Working venom solution containing 25 µg/10 µl was prepared using the same buffer.

#### Anti-snake venom

Lyophilized polyvalent ASV was procured from Bharat Serums and Vaccines Pvt. Ltd. Ambarnath, Maharashtra, India. Contents of the entire vial was dissolved in 10 ml sterile water provided by the manufacturer and stored at 2–8 °C. As per manufacturer’s data, each ml of the reconstituted ASV was capable of neutralizing 0.6 mg of N.N venom. Total protein content in ASV was determined to be 2.2 mg/ml by Lowry’s method (Lowry et al. 1951). Three different aliquots of reconstituted ASV viz. 25 µl, 42 µl and 60 µl containing 55 µg, 92.4 µg and 132 µg protein respectively were used for the assay. According to manufacturer’s data, 42 µl ASV equivalent to 92.4 µg protein is capable of neutralizing 25 µg N.N venom. To assess the efficacy of ASV in rescuing the A2MG inactivation, ASV was used over a range of concentrations (25 to 60 µl).

#### Methanolic extract of Andrographis paniculata (MAP)

MAP was obtained from Natural Remedies Pvt. Ltd. Bangalore, Karnataka, India. Three different stock solutions of MAP were prepared by dissolving 2.5 mg, 5 mg and 10 mg each in 1 ml of dimethyl sulfoxide. From each stock solution, 10 µl containing 25 µg, 50 µg and 100 µg were used for the assay.

GC–MS analysis was carried out at Analytical Research and Metallurgical Laboratories Pvt. Ltd. Bangalore, Karnataka, India, which revealed the presence of 44 phytocompounds in MAP (Nayak et al. 2020a).

Qualitative analysis of MAP showed the presence of terpenoids, phenols, tannins, flavonoids and carbohydrates. Quantitative analysis demonstrated the total phenolic and flavonoid content as 43.55 mg gallic acid equivalents/g and 11 mg quercetin equivalents/g respectively in MAP (Nayak et al. 2020).

#### Methodology

A2MG activity of heparinized human plasma was estimated based on the chymotrypsin-bound esterase (CTBE) activity as described by Rao et al. (1984) with modifications.

**Fig. 1:**
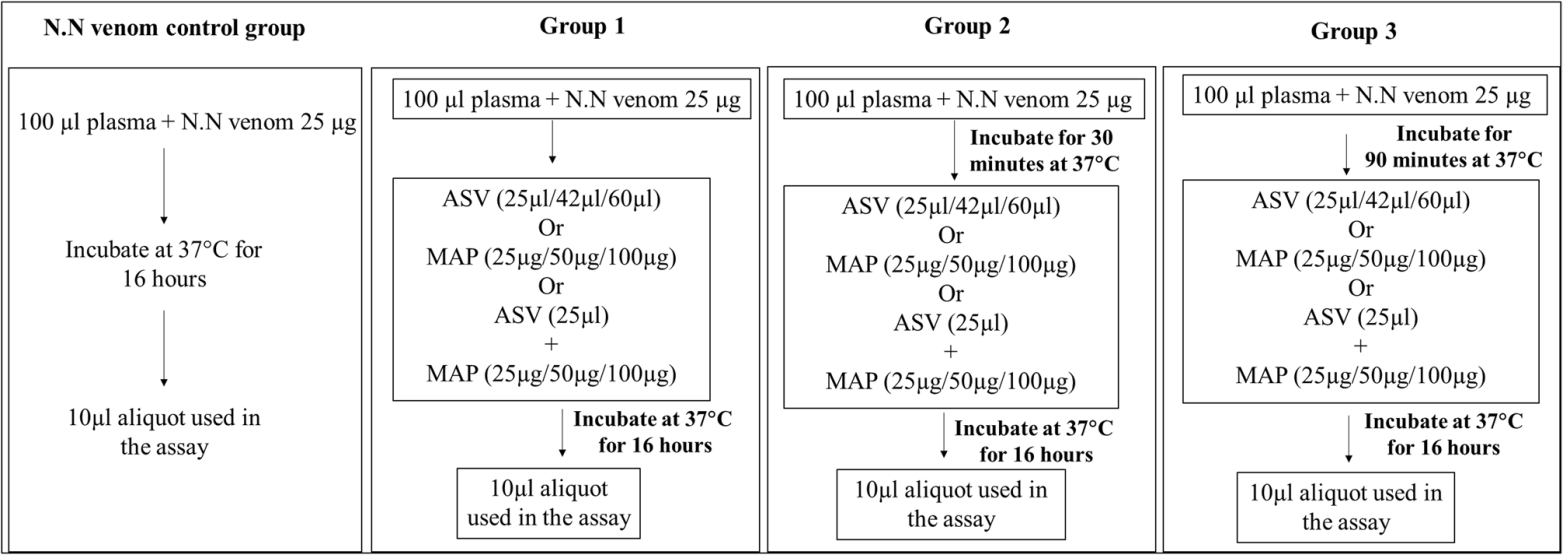
Flow chart representing the details of the in-vitro experiments conducted during the estimation of effects of various concentrations of ASV, MAP and ASV + MAP on A2MG activity in different groups. N.N-*Naja naja*; ASV-anti-snake venom; MAP-methanolic extract of *Andrographis paniculata*;

A2MG activity of plasma in the presence of N.N venom was estimated by pre-incubating plasma (0.1 ml) with N.N venom (25 µg) at 37 °C for 16 h (N.N venom Control, Fig. 1), following which 10 µl aliquot was used for the estimation of A2MG. Effect of ASV and MAP on A2MG activity was studied using various concentrations of ASV (25–60 µl equivalent to 55–132 µg protein by Lowry’s method) and MAP (25–100 µg). Effect of combination of ASV and MAP was studied using a reduced concentration of 25 µl of ASV and 25–100 µg of MAP. The experiments were carried out in 3 groups.

**Group 1 experiments:** Plasma was mixed with NN venom followed by the immediate addition of ASV, MAP, ASV + MAP and mixture was incubated for 16 h at 37 °C and A2MG activity was estimated (Fig. 1). Though this was an in vitro experiment, this group represents the situation where a snake bite victim is immediately treated for envenomation.

**Group 2 experiments:** Plasma was incubated with N.N venom for 30 min at 37 °C, followed by the addition of ASV or MAP or ASV + MAP. Further, the reaction mixture was incubated at 37 °C for 16 h to enable the interaction of antibodies in ASV and phytoconstituents of MAP with each other and with A2MG (Fig. 1). Following this A2MG activity was estimated. This group represents a situation where a snake bite victim is treated after a time lapse of 30 min after the bite.

**Group 3 experiments:** Plasma was incubated with N.N venom for 90 min at 37 °C, followed by the addition of ASV or MAP or ASV + MAP. The reaction mixture was then incubated at 37 °C for 16 h and A2MG activity was measured (Fig. 1). The time lapse of 90 min was specifically chosen since in this period, the ‘ptosis of the eyes’, which is the first sign of neurotoxicity in N.N envenomation is likely to appear, after which treatment with polyvalent ASV is usually initiated.

### In-vivo studies

#### Animals

The study was conducted after obtaining approval from Institutional Animal Ethics Committee (IAEC/KMC/26/2017). Healthy female Wistar rats (*n* = 115) weighing between 180–200 g and Swiss albino mice (*n* = 15) weighing between 18–20 g were used for the study. Animals were bred in the central animal house of Manipal Academy of Higher Education, Manipal. Maintenance of animals was according to the guidelines of Committee for the Purpose of Control and Supervision of Experiments on Animals (CPCSEA) and animal welfare division, Government of India. All the animals were housed in polypropylene cages at 28 ± 1 °C and 50 ± 5% humidity, with two animals in each cage. Animals were fed with standard laboratory feed (Gold Mohur, Lipton India Ltd) and water ad libitum.

#### *Naja naja* venom

Stock solution of N.N venom was prepared by dissolving 10 mg venom in 1 ml of saline (0.9%) and stored at 4 °C.

#### Calculation of median lethal dose (LD_50_) of N.N venom and its administration to female Wistar rats

Median lethal dose (LD_50_) of N.N venom was calculated using OECD guidelines 425 and software AOT 425StatPgm. According to this, different concentrations of N.N venom were administered intramuscularly (i.m) to female Wistar rats (*n* = 15) weighing about 180–200 g. Initially, venom concentration of 2000 mg/Kg body weight of the rat was administered, following which the dose for each successive animal was adjusted down by a factor of 3.2. All the animals were observed initially for 30 min, 4 h and after that, daily for 14 days. General behavior and number of deaths in 24 h were noted. LD_50_ was calculated using AOT 425 statistical program. N.N venom at LD_50_ concentration was administered i.m into the left thigh muscle of rats for efficacy studies using ASV and MAP.

#### Preparation of ASV

Reconstitution of the commercially available lyophilized polyvalent ASV is mentioned under in-vitro materials. Dosage of ASV was calculated based on manufacturer’s instructions (1 ml of reconstituted ASV can neutralize 0.6 mg of N.N venom) and administered intraperitoneally to animals after injecting LD_50_ venom.

#### Dose fixation study for determination of effective dose (ED) of MAP

Effective dose of MAP was determined in Swiss albino mice (*n* = 15). Animals were randomly divided into 5 groups of 3 animals each. Group 1 received 0.25% w/v carboxymethylcellulose (CMC) orally (p.o) and served as vehicle control. Group 2 received lethal dose (LD_99_) of the venom intramuscularly (i.m) i.e. 0.14 mg/kg body weight (b.w) and served as venom control. Group 3, 4 and 5 received 3 different doses of MAP [100, 200 and 400 mg/Kg b.w orally, after the administration of LD_99_ venom (i.m). All the animals were observed initially for 30 min, 4 h and after that, daily for 14 days. Number of deaths were noted. The dose of MAP at which maximum survival was observed was designated as ED and was used for further efficacy studies. These data obtained with the dose fixation study in Swiss albino mice was carried out to restrict the wastage of resources and used to calculate the ED in female Wistar rats.

#### Experimental design for efficacy study: (Fig. 2)

**Fig. 2:**
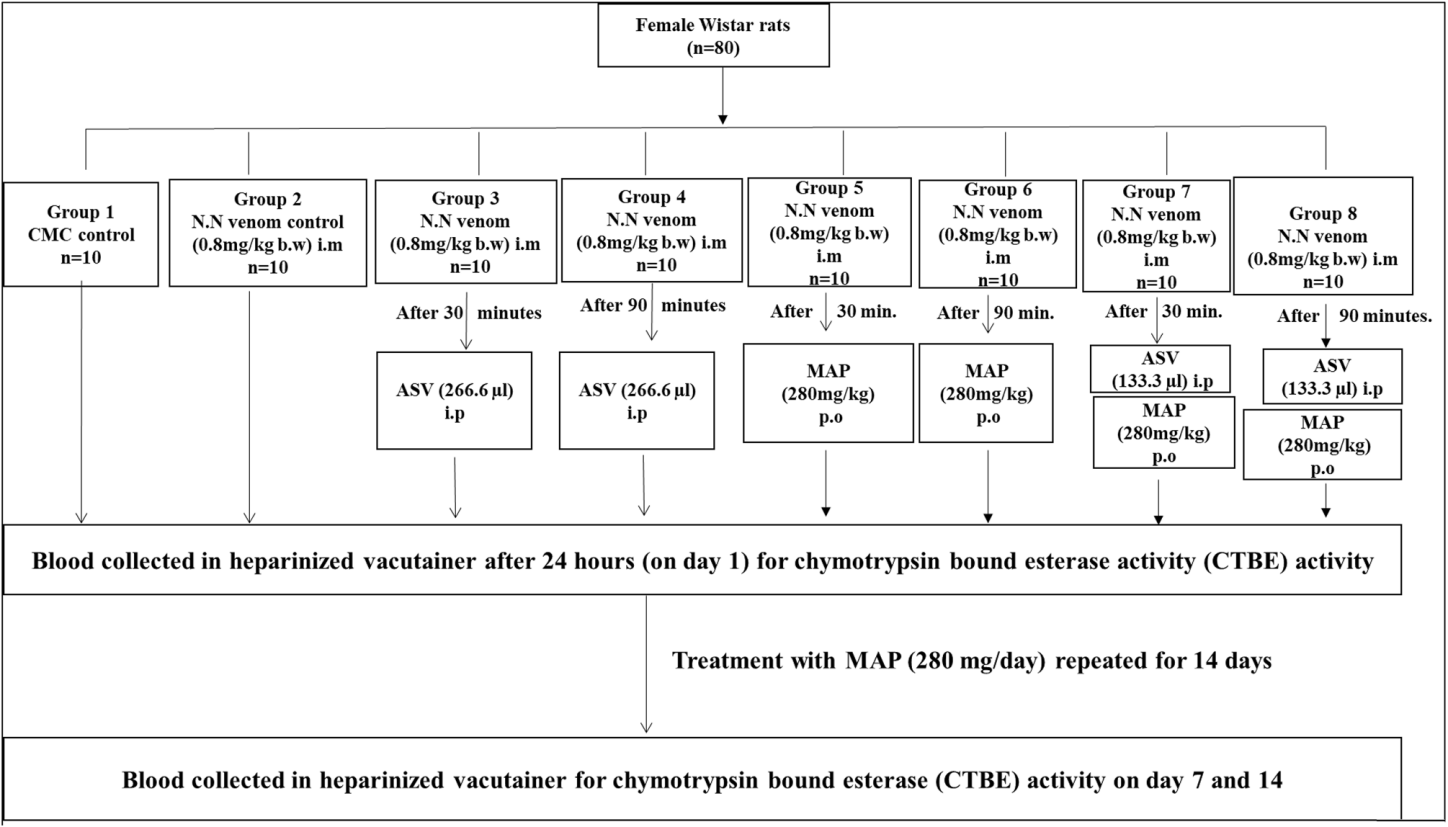
Flowchart representing the methodology for efficacy study using polyvalent ASV, MAP and their combination on alterations in chymotrypsin bound esterase (CTBE) activity induced by *Naja naja* venom in female Wistar rats. *CMC* carboxymethylcellulose, *LD*_*50*_ median lethal dose, *N.N* *Naja naja*, *b.w* body weight, *ASV* anti-snake venom, *MAP* methanolic extract of *Andrographis paniculata*, *i.m* intramuscular, *i.p* intraperitoneal, *p.o* per oral

The study included 8 groups (*n* = 80) of female Wistar rats having 10 rats in each group. Group 1 received CMC (0.25%) orally and served as vehicle control. Group 2 served as N.N venom control, where N.N venom at LD_50_ concentration was administered i.m into the left thigh muscle. Group 3 to Group 8 were treatment groups. In these groups treatment with ASV/MAP/ASV + MAP was once again administered 30 or 90 min after the administration of venom at LD_50_ for reasons as mentioned for the in-vitro experiments. Group 3 and 4 received i.p administration of single dose of ASV (266.6 µl) after 30 min and 90 min respectively, after administration of N.N venom (i.m) at LD_50_ concentration. Dosage of ASV used in the study was according to the manufacturer’s instructions as mentioned under ‘Materials and methods’. Group 5 and 6 received MAP at ED orally, 30 and 90 min respectively after the administration of N.N venom at LD_50_. A reduced dosage of ASV (50% reduction compared to Group 3 and 4)) was administered to Group 7 and 8 along with supplementation with MAP at ED after 30 and 90 min of venom administration respectively. Blood was collected from retro-orbital plexus in heparinized vacutainers after 24 h. Plasma was separated and used for the estimation of chymotrypsin bound esterase (CTBE) activity contributed by all the 3 macroglobulin homologues. For Group 5 to 8, treatment with MAP was continued at the same dose for 14 days. Estimation of CTBE activity was repeated on day 7 and 14 in all the groups. Number of animals surviving in each group was noted.

### Statistical analysis

Data was analyzed using SPSS 16.0. For in-vitro studies, A2MG activity was represented in terms of Mean ± SEM for replicate samples (*n* = 3). One-way ANOVA followed by Tukey’s post hoc test was used to determine the significance between the groups. *p* ≤ 0.05 considered statistically significant.

For in-vivo studies, the number of animals surviving in each group was represented as percentage. Results were represented as Mean ± SD of CTBE activity in each group. R-ANOVA followed by Tukey’s post hoc test was used to determine the significance within the group and between the groups, *p* ≤ 0.05 being considered statistically significant.

## Results

### In-vitro experiments: (Table 1)

**Table 1:** In-vitro analysis of the effects of ASV, MAP and ASV + MAP in preventing the inactivation of A2MG activity by *Naja naja* venom

	Group 1 A2MG activity (mg/dl) Mean ± SEM	Group 2 A2MG activity (mg/dl) Mean ± SEM	Group 3 A2MG activity (mg/dl) Mean ± SEM
Plasma control (P)	307 ± 2.68	307 ± 2.68	307 ± 2.68
P + N.N venom (25 µg) [Venom Control]	138.7 ± 2.0 ^@^ (*p* = 0.001)	138.7 ± 2.0 ^@^ (*p* = 0.001)	138.7 ± 2.0 ^@^ (*p* = 0.001)
P + N.N venom (25 µg) + ASV (25 µl)	228.8 ± 7.47 ^#^ (*p* = 0.001)	177.72 ± 0.8 ^#^ (*p* = 0.001)	167.55 ± 1.78 ^#^ (*p* = 0.001)
P + N.N venom (25 µg) + ASV (42 µl)	267.27 ± 2.7 ^#,*^ (*p* = 0.001)	205.09 ± 0.9 ^#,*^ (*p* = 0.001)	184.46 ± 1.56 ^#,*^ (*p* = 0.001)
P + N.N venom (25 µg) + ASV (60 µl)	282.8 ± 2.1 ^#,*^ (*p* = 0.001)	225.07 ± 2.59 ^#,*^ (*p* = 0.001)	216.19 ± 2.79 ^#,*^ (*p* = 0.001)
P + N.N venom (25 µg) + MAP (25 µg)	201.06 ± 2.1 ^#,*^ (*p* = 0.001)	172.54 ± 2.41 ^#^ (*p* = 0.001)	162.74 ± 2.97 ^#^ (*p* = 0.001)
P + N.N venom (25 µg) + MAP (50 µg)	234.13 ± 3.75 ^#^ (*p* = 0.001)	199.18 ± 0.6 ^#,*^ (*p* = 0.001)	177.17 ± 0.66 ^#^ (*p* = 0.001)
P + N.N venom (25 µg) + MAP (100 µg)	260.02 ± 2.08 ^#,*^ (*p* = 0.001)	230.46 ± 5.1 ^#,*^ (*p* = 0.001)	213.79 ± 1.44 ^#,*^ (*p* = 0.001)
P + N.N venom (25 µg) + ASV (25 µl) + MAP (25 µg)	237.85 ± 2.37 ^#^ (*p* = 0.001)	189.38 ± 1.44 ^#^ (*p* = 0.001)	175.5 ± 2.49 ^#^ (*p* = 0.001)
P + N.N venom (25 µg) + ASV (25 µl) + MAP (50 µg)	270.93 ± 3.75 ^#,*^ (*p* = 0.001)	209.35 ± 2.25 ^#,*^ (*p* = 0.001)	189.19 ± 3.37 ^#,*^ (*p* = 0.001)
P + N.N venom (25 µg) + ASV (25 µl) + MAP (100 µg)	285.91 ± 3.39 ^#,*^ (*p* = 0.001)	240.97 ± 3.0 ^#,*^ (*p* = 0.001)	220.6 ± 0.97 ^#,*^ (*p* = 0.001)

Group 1: Estimation of A2MG activity where, ASV or MAP or ASV + MAP was added immediately to plasma containing N.N venom and the reaction mixture was incubated at 37 °C for 16 h. Group 2: Estimation of A2MG activity where, ASV or MAP or ASV + MAP was added after 30 min of incubation of N.N venom with plasma following which the reaction mixture was incubated at 37 °C for 16 h. Group 3: Estimation of A2MG activity where, ASV or MAP or ASV + MAP was added after 90 min of incubation of N.N venom with plasma following which the reaction mixture was incubated at 37 °C for 16 h. Where ^@^*p* < 0.05 compared to plasma control (P); ^#^*p* < 0.05 compared to Venom control; where^*^p < 0.05 compared to P + N.N venom (25 µg) + ASV (25 µl)

*A2MG* α_2_-macroglobulin, *N.N* *Naja naja*, *ASV* polyvalent anti-snake venom, *MAP* methanolic extract of Andrographis paniculata

**Fig. 3:**
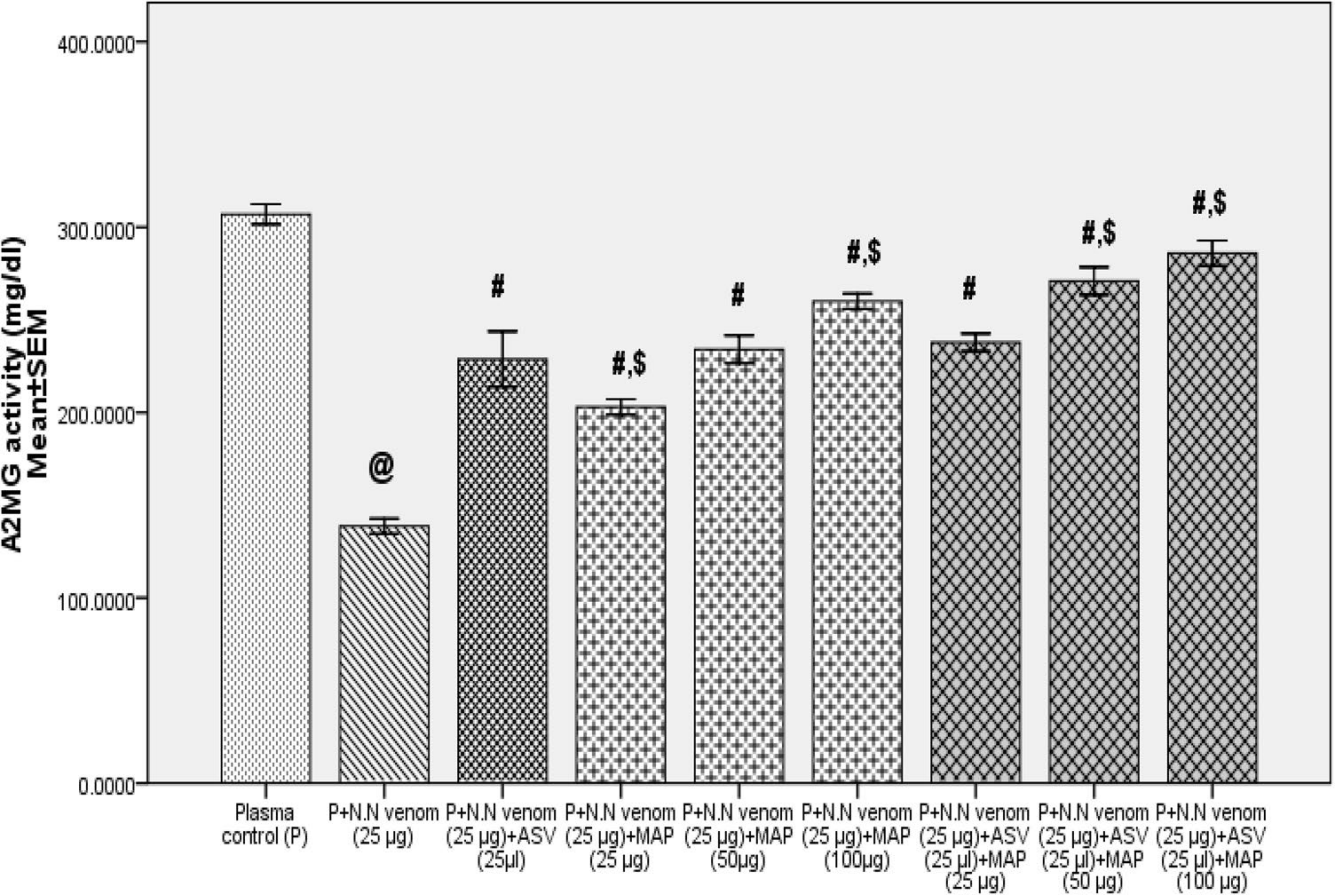
Comparison of effects of ASV, MAP and ASV + MAP on *Naja naja* venom induced inactivation of A2MG activity in Group 1. Group 1: Estimation of A2MG activity where, ASV or MAP or ASV + MAP were added immediately to plasma containing N.N venom and the reaction mixture was incubated at 37 °C for 16 h. Where^@^*p* < 0.05 compared to plasma control, ^#^*p* < 0.05 compared to P + N.N venom, ^$^*p* < 0.05 compared to P + N.N venom + ASV (25 µl). *A2MG* α_2_-macroglobulin, *N.N* *Naja naja*, *ASV* polyvalent anti-snake venom, *MAP* methanolic extract of *Andrographis paniculata*

Addition of N.N venom (25 µg) to human plasma and immediate estimation of A2MG did not cause any loss of activity. Incubation of plasma with venom for 16 h at 37 °C caused a significant decrease (55%, *p* = 0.001) in A2MG activity compared to plasma control. Immediate addition of ASV ((25 to 60 µl) to plasma containing N.N venom showed a concentration dependent rescue of A2MG activity to the extent of 65 to 100% respectively in Group 1 Experiments. When plasma was incubated with venom and MAP (25 to 100 µg), A2MG activity was rescued to the extent of 46 to 88%. When ASV concentration was reduced to 25 µl, and supplemented with MAP (25 to 100 µg), the A2MG activity was rescued to the extent of 72 to 100% compared to N.N venom control. The rescue of A2MG was more by 4 to 25% than when ASV (25 µl) was used alone (*p* = 0.001) (Fig. 3), confirming a very effective supplementation of low concentrations of ASV by MAP.

**Fig. 4:**
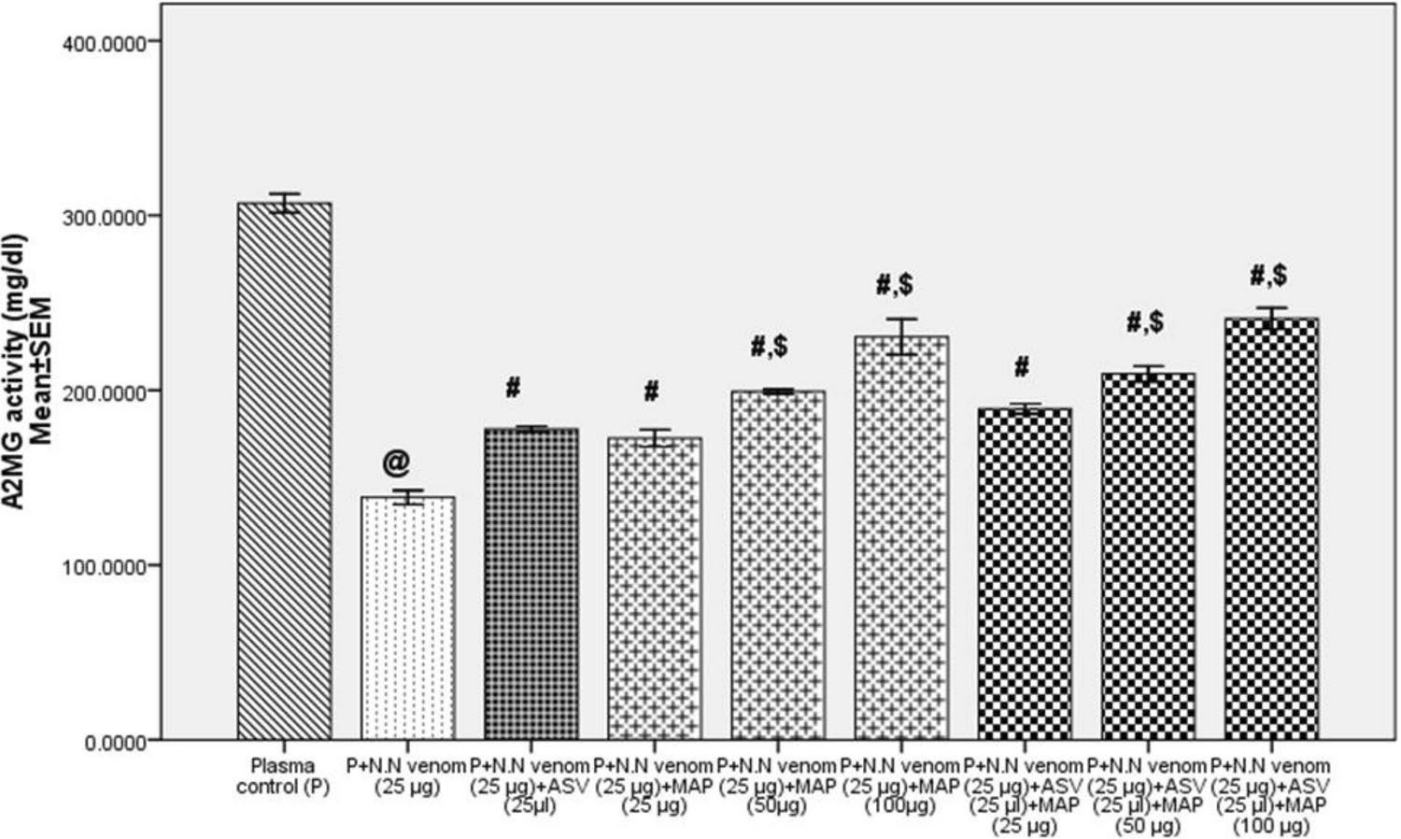
Comparison of effects of ASV, MAP and ASV + MAP on *Naja naja* venom induced inactivation of A2MG activity in Group 2. **Group 2:** Estimation of A2MG activity where, ASV, MAP and ASV + MAP were added after 30 min of incubation of N.N venom with plasma following which the reaction mixture was incubated at 37 °C for 16 h, where ^@^*p* < 0.05 compared to plasma control, ^#^*p* < 0.05 compared to P + N.N venom, ^$^*p* < 0.05 compared to P + N.N venom + ASV (25 µl). *A2MG* α_2_-macroglobulin, *N.N Naja naja* , *ASV* polyvalent anti-snake venom, *MAP* methanolic extract of *Andrographis paniculata*

In Group 2, N.N venom was allowed to react with plasma for 30 min prior to the addition of ASV or MAP or their combination. ASV (25 to 60 µl) was able to rescue A2MG to the extent of 28 to 62% (*p* = 0.001) which was 37–38% less than in Group 1 experiments. MAP (25 to 100 µg) was able to reverse the inactivation of A2MG to the extent of 24 to 66% (*p* = 0.001), which was 22% less than in Group 1 experiments. Rescue of A2MG activity was up to 74% (*p* = 0.001) when ASV (25 µl) was supplemented with MAP (25 to 100 µg) (Fig. 4) which was 26% less than in Group 1 experiments.

**Fig. 5:**
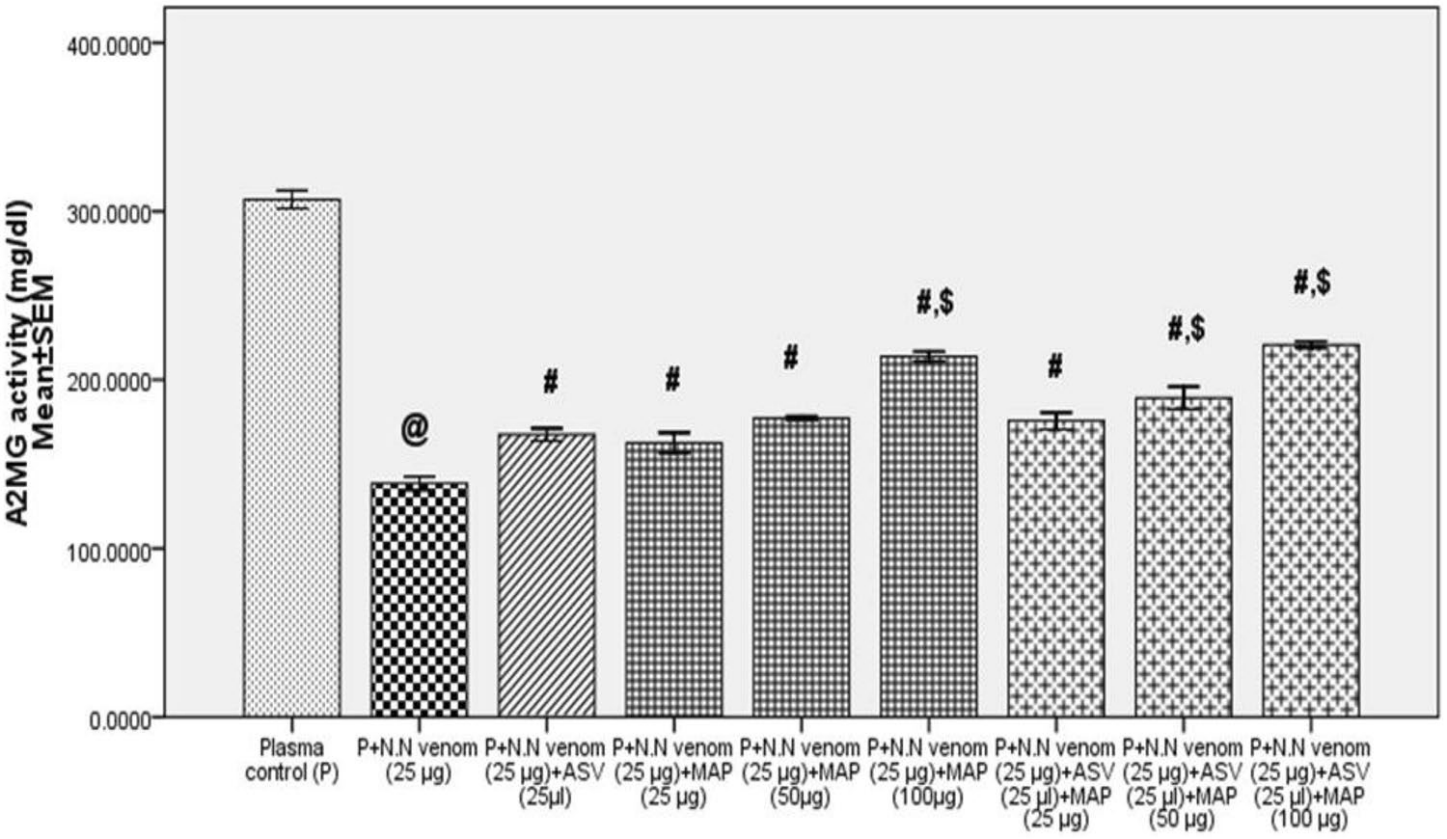
Comparison of effects of ASV, MAP and ASV + MAP on *Naja naja* venom induced inactivation of A2MG activity in Group 3. Group 3: Estimation of A2MG activity where, ASV, MAP and ASV + MAP was added after 90 min of incubation of N.N venom with plasma following which the reaction mixture was incubated at 37 °C for 16 h. Where ^@^*p* < 0.05 compared to plasma control, where ^#^*p* < 0.05 compared to P + N.N venom, ^$^*p* < 0.05 compared to P + N.N venom + ASV (25 µl). *A2MG* α_2_-macroglobulin, *N.N* Naja naja; *ASV* polyvalent anti-snake venom, *MAP* methanolic extract of *Andrographis paniculata*

In Group 3 experiments, N.N venom was allowed to react with plasma for 90 min prior to the addition of ASV or MAP or their combination. ASV and MAP when used individually or in combination prevented the inactivation of A2MG to a significant extent (*p* = 0.001) of 21 to 56%, 17 to 54% and 27 to 59% respectively (Fig. 5). This was 44%, 29–34% and 45% less compared to Group 1 experiments and 6–7%, 7–8% and 10–15% less than in Group 2 experiments.

### In-vivo experiments

LD_50_ of N.N venom was determined to be 0.8 mg/kg body weight (b.w) of rats as per AOT 425 StatPgm software. N.N venom at LD_50_ concentration (0.8 mg/kg b.w) was used for efficacy studies where ASV, MAP and their combination was used. MAP at 100 and 200 mg/kg b.w, could not protect the mice from death. However, ED of MAP was found to be 400 mg/kg b.w in albino mice at which maximum survival (2 out of 3) was observed (Table 2). This dose was calibrated to the rat dose i.e.280 mg/kg b.w (Paget, G. E. and Barnes, 1964) and was used for efficacy studies using MAP and its combination with ASV in female Wistar rats.

**Table 2:** Effects of ASV, MAP and ASV + MAP on N.N venom induced mortality in female Wistar rats

	Mortality rate (number of death/number of animals used)	Percentage survival as on Day 14 (%)
**Group 1** CMC control (0.25%)	0/10	100
**Group 2** LD_50_ N.N venom (0.8 mg/kg)	4/10	60
**Group 3** LD_50_ N.N venom + ASV (266.6 µl) injected after 30 min	1/10	90
**Group 4** LD_50_ N.N venom + ASV (266.6 µl) injected after 90 min	1/10	90
**Group 5** LD_50_ N.N venom + MAP (280 mg/kg) administered after 30 min	2/10	80
**Group 6** LD_50_ N.N venom + MAP (280 mg/kg) administered after 90 min	3/10	70
**Group 7** LD_50_ N.N venom + ASV (133.3 µl) + MAP (280 mg/kg) administered after 30 min	1/10	90
**Group 8** LD_50_ N.N venom + ASV (133.3 µl) + MAP (280 mg/kg) administered after 90 min	1/10	90

*N.N* Naja naja, *ASV* anti-snake venom, *MAP* methanolic extract of *Andrographis paniculata*, *LD*_*50*_ median lethal dose

**Fig. 6:**
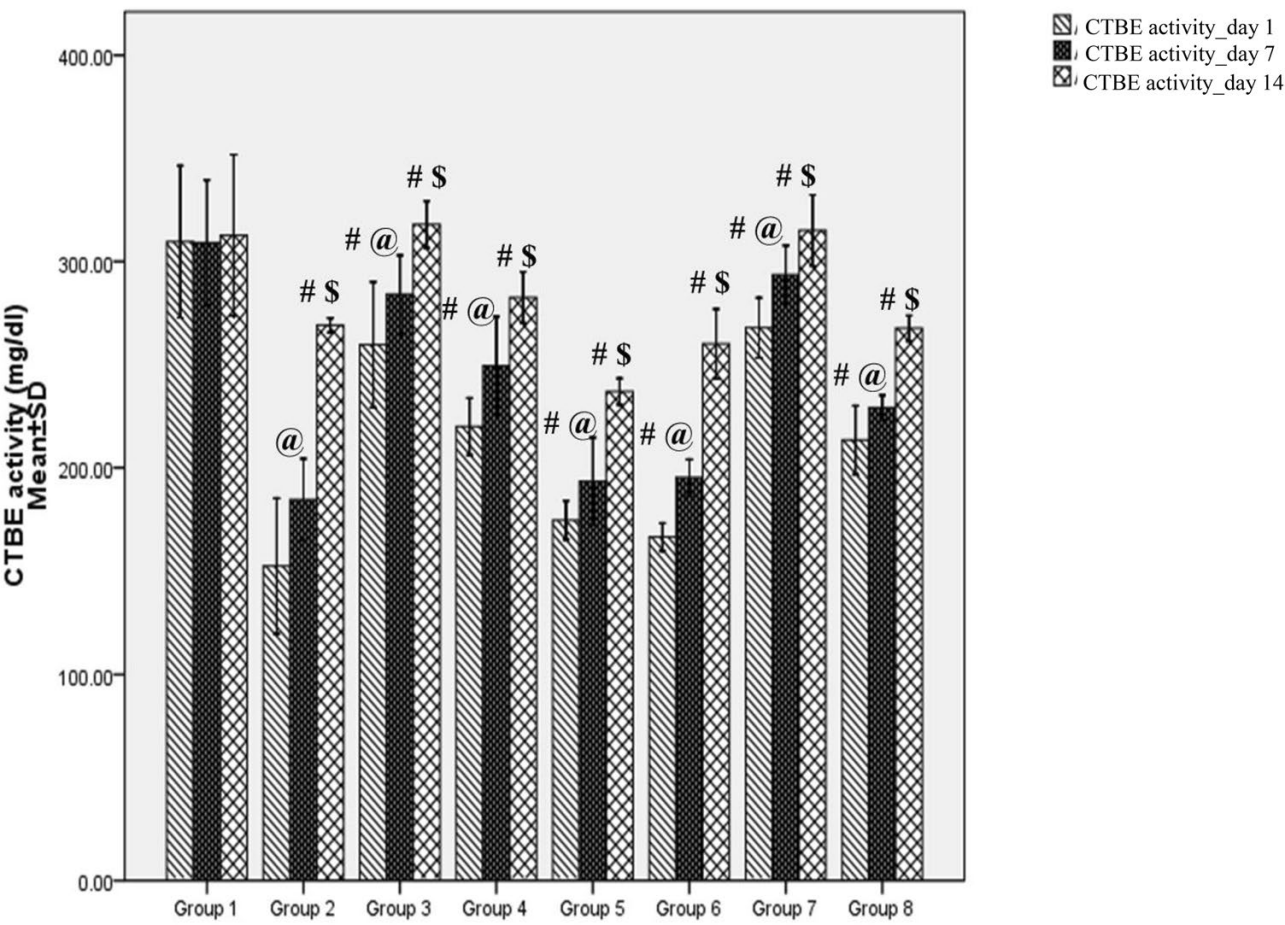
Effects of ASV, MAP and ASV + MAP in preventing the inactivation of A2MG activity by *Naja naja* venom in female Wistar rats. Group 1: CMC control p.o (0.25%); Group 2: LD_50_ N.N venom i.m (0.8 mg/kg b.w);Group 3: LD_50_ N.N venom i.m (0.8 mg/kg b.w) + ASV (266.6 µl) i.p injected after 30 min of N.N venom; Group 4: LD_50_ N.N venom i.m (0.8 mg/kg b.w) + ASV (266.6 µl) i.p injected after 90 min of N.N venom; Group 5: LD_50_ N.N venom i.m (0.8 mg/kg b.w) + MAP (280 mg/kg b.w) p.o administered after 30 min of N.N venom; Group 6: LD_50_ N.N venom i.m (0.8 mg/kg b.w) + MAP (280 mg/kg b.w) p.o administered after 90 min of N.N venom; Group 7: LD_50_ N.N venom i.m (0.8 mg/kg b.w) + ASV (133.3 µl) i.p + MAP (280 mg/kg b.w) p.o administered after 30 min of N.N venom; Group 8: LD_50_ N.N venom i.m (0.8 mg/kg b.w) + ASV (133.3 µl) i.p + MAP (280 mg/kg b.w) p.o administered after 90 min of N.N venom. *CMC* carboxymethylcellulose, *LD*_*50*_ median lethal dose, *N.N* *Naja naja*, *b.w* body weight, *ASV* anti-snake venom, *MAP* methanolic extract of *Andrographis paniculata*, *i.m* intramuscular, *i.p* intraperitoneal, *p.o* per oral

CTBE activity in rat plasma was found to be 309–312 mg/dl in Group 1 (control). This represents the total activity of all the 3 macroglobulin homologues. N.N venom at LD_50_ caused 40% death in Group 2 (Table 3) and showed significant decrease (51%, *p* = 0.001) in CTBE activity compared to Group 1, estimated after 24 h of venom administration (day 1) (Table 3). CTBE activity improved by 21% and 76% on day 7 and 14 respectively. A single dose of ASV showed 90% survival in both Group 3 and 4 (Table 3). In Group 3, ASV prevented the inactivation of CTBE activity to an extent of 70% (*p* = 0.001) compared to Group 2 on day 1 and the activity was completely restored on day 14. In Group 4, where ASV was administered after 90 min of envenomation, inactivation of CTBE activity was prevented to an extent of 44% (*p* = 0.001) on day 1 (Table 3). Further improvement of CTBE activity was up to 29%, when measured on day 14. Oral administration of repeated doses of MAP decreased the mortality by 30% compared to Group 2. MAP showed up to 15% improvement in CTBE activity compared to Group 2 on day 1. When treatment with MAP was continued for 14 days, CTBE activity was improved up to 56% compared to day 1. When ASV was reduced by 50% and supplemented with ED of MAP, a 90% survival was observed both in Group 7 and 8. Loss of CTBE activity was prevented to an extent of 76% (*p* = 0.001) and 40% (*p* = 0.001) in Group 7 and 8 respectively, compared to Group 2 on day 1 (Fig. 6). Further treatment with MAP at ED for 14 days, completely restored CTBE activity in Group 7 (Fig. 6).

**Table 3:** Effect of polyvalent ASV, MAP and their combination on alterations in chymotrypsin bound esterase (CTBE) activity induced by *Naja naja* venom in female Wistar rats

	CTBE activity (mg/dl) Day-1 Mean ± SD	CTBE activity (mg/dl) Day-7 Mean ± SD	CTBE activity (mg/dl) Day-14 Mean ± SD
Group 1 CMC control (0.25%)	309.58 ± 18.4	309.1 ± 15.1	312.6 ± 19.6
Group 2 LD_50_ N.N venom (0.8 mg/kg)	152.57 ± 16.4 ^*^ (*p* = 0.001)	184.7 ± 9.8 ^*^ (*p* = 0.001)	269.1 ± 1.7 ^*^ (*p* = 0.001)
Group 3 LD_50_ N.N venom + ASV (266.6 µl) injected after 30 min	259.6 ± 15.2 ^**^ (*p* = 0.001)	284.08 ± 9.4 ^**^ (*p* = 0.001)	317.9 ± 5.7 ^**^ (*p* = 0.001)
Group 4 LD_50_ N.N venom + ASV (266.6 µl) injected after 90 min	220 ± 6.9 ^**^ (*p* = 0.001)	249.4 ± 11.9 ^**^ (*p* = 0.001)	282.6 ± 6.2
Group 5 LD_50_ N.N venom + MAP (280 mg/kg) administered after 30 min	174.7 ± 4.7	193.6 ± 10.5	236.9 ± 3.2 ^**^ (*p* = 0.005)
Group 6 LD_50_ N.N venom + MAP (280 mg/kg) administered after 90 min	166.6 ± 3.6	195.3 ± 4.3	260 ± 8.5
Group 7 LD_50_ N.N venom + ASV (133.3 µl) + MAP (280 mg/kg) administered after 30 min	267.8 ± 7.3 ^**^ (*p* = 0.001)	293.1 ± 7.0 ^**^ (*p* = 0.001)	315 ± 8.6 ^**^ (*p* = 0.001)
Group 8 LD_50_ N.N venom + ASV (133.3 µl) + MAP (280 mg/kg) administered after 90 min	213.5 ± 8.3 ^**^ (*p* = 0.001)	229.1 ± 3.0 ^**^ (*p* = 0.001)	267.6 ± 3.1

*N.N* *Naja naja*, *ASV* polyvalent anti-snake venom, *MAP* methanolic extract of *Andrographis paniculata*, *CMC* carboxymethyl cellulose, *LD*_*50*_ median lethal dose

## Discussion

In in-vitro experiments, using human plasma, addition of venom to plasma and incubation up to 90 min followed by the estimation of A2MG did not cause any loss of activity of A2MG, which proves that inactivation of A2MG requires interaction with venom enzymes for a prolonged period of time as observed earlier (Roche and Pattabiraman 1990). In the reaction mixture where there are antibodies specific to N.N venom enzymes and MAP constituents with proven anti-venom properties (Sivakumar and Manikandan 2015), there is bound to be an intense competition between these participants for binding/interacting with A2MG, though their mechanisms of interaction are bound to be different. Venom enzymes which are not neutralized by either the antibodies or MAP, need to gain access to the ‘bait’ region of A2MG and cleave it, to be trapped inside. Therefore, it is safe to assume that inactivation of A2MG observed in the presence of ASV and MAP is due to enzymes which escape both these modes of inhibition. The fact that addition of ASV was capable of substantial rescue of A2MG, in all the test groups, demonstrates that ASV contains appropriate antibodies to neutralize A2MG inactivating enzymes. However, the polyvalent ASV was maximally effective at a concentration which was 42% higher (with regard to rescue of A2MG) than advised by the manufacturer. Earlier studies which investigated the efficacy of ASV in inhibiting AChE and hyaluronidase activity of N.N venom demonstrated that efficacy of ASV was limited to the extent of 23% and 41% (Nayak et al. 2020). A thromboelastographic study which investigated the effectiveness of ASV in normalizing hemostatic abnormalities induced by N.N venom revealed a 38% higher requirement of ASV than advised by the manufacturer (Nayak et al. 2020a). This highlights the fact that, the efficacy of ASV varies against different parameters of toxicity in the venom.

In case of N.N envenomation, ASV is administered when the first sign of neurotoxicity, i.e., the ‘ptosis of the eyes’ appears. Clinicians treating envenomation, normally rely largely on reversal of symptoms as a guideline for adjusting the dosage of ASV. Perhaps it is also important to calibrate the recovery of the patient against the improvement in A2MG activity in plasma, in addition to other routine blood tests such as prothrombin time and international normalized ratio. When venom enzymes had adequate time (30 or 90 min), to diffuse into the ‘trap’ of A2MG to inactivate it, a lower rescue of A2MG by ASV was observed. This is because of the partial irreversible inactivation of A2MG by venom enzymes. MAP was almost as effective as ASV when used individually in rescuing A2MG, which can be attributed to the presence of plant metabolites such as flavonoids, terpenoids, polyphenols, tannins and phenolic compounds (Sivakumar and Manikandan 2015; Nayak et al. 2020), which bind to the active sites of enzymes as demonstrated by ‘in silico’ experiments (Sivakumar and Manikandan 2015). It is also possible that small molecular weight (148 to 380 Da) MAP constituents such as isopropyl phenyl ketone, cyclopentane, 1-dodecene, 1-pentadecene, phytol, glycidol stearate and coprostanol (Nayak et al. 2020) might be capable of penetrating the ‘trap’ of A2MG, preventing the binding of venom enzymes to A2MG, thereby preventing loss of activity. These enzymes which are prevented from binding to A2MG would be targets for neutralization by ASV and larger MAP constituents. N.N venom also contains significant amounts of L-amino acid oxidase which generates hydrogen peroxide (H_2_O_2_) which is known to inactivate A2MG (Choudhury et al. 2017). H_2_O_2_ is also a precursor for more powerful free radicals such as hydroxyl radical and hypochlorous acid which are known to be potent inactivators of A2MG (Siddiqui et al. 2016). Since MAP contains high concentrations of antioxidant molecules (Dey et al. 2013; Das and Srivastav 2014), it can neutralize H_2_O_2_, preventing oxidative damage (Sheeja et al. 2006) and buttressing A2MG activity. This is probably the reason why supplementing low concentrations of ASV with MAP showed a much better effect than when ASV or MAP was used alone. This corroborates with findings of previous experiments on other parameters of N.N venom toxicity which were also mitigated by this multi-pronged strategy (Nayak et al. 2020; Nayak et al. 2021). Maintaining higher effective concentrations of ASV by preventing the oxidative damage of antibodies in ASV is also a likely mechanism operating here.

In in-vivo studies using envenomed rats, the venom enzymes target the macroglobulin homologues. This is reflected by the low CTBE activity in plasma following envenomation on day 1. CTBE activity progressively improved by 21% and 76% on day 7 and 14 respectively which can be attributed to the role of the liver in the re-synthesis of these proteins. Decrease in mortality on administration of ASV as per manufacturer’s instructions, proved that ASV was effective to the extent of 90%. A single dose of ASV was able to rescue CTBE activity in a time-dependent manner, being more efficient when administered early. Clinical studies in envenomed patients have repeatedly highlighted the importance of early treatment of poisonous snake bites to improve the outcome. After treatment with ASV, complete restoration of CTBE activity on day 14 was recorded which can also be due to the role of liver in the re-synthesis of macroglobulin homologues.

MAP alone was 10% less effective than ASV in reducing mortality. MAP showed a minimal effect on rescue of CTBE activity in rats when measured on day 1, in contrast to in-vitro experiments. However, it was surprising to note that supplementation of a single reduced dose (50% reduction) of ASV followed by repeated doses of MAP up to 14 days, not only improved the survival rate, but also rescued the CTBE activity completely. This may be due to the hepatoprotective activity of MAP constituents (Nagalekshmi et al. 2011; M 2013). The results from this study strongly support the view that the efficacy of ASV can be enhanced in the presence of MAP. Triggering antibody production (Alam and Gomes 1998) in the rat, chelation of metal ions and inhibition of metalloenzymes of N.N venom (Castro et al. 1999), antioxidant action (M 2013) leading to prevention of inactivation of macroglobulin homologues are some of the likely mechanisms involved in toxin neutralization and add further dimensions to the multipronged strategy employed here.

## Conclusion

The study investigated the efficacy of the standard polyvalent ASV in rescuing human A2MG (in-vitro) and its homologues in the rat (in-vivo), when exposed to N.N venom and compared it to that of MAP. It also tested a novel strategy of supplementation of ASV with MAP. In humans, use of the Indian ASV is fraught with perils. Its inadequacy in addressing the toxicity of the multiple toxins of low molecular weight and low immunogenicity is well documented. The finding that supplementation of low doses of ASV with MAP, not only improved survival in rats, but also augmented the rescue of A2MG homologues, has wide implications in the treatment of envenomation, since it restores the role of these proteinase inhibitors as ‘physiological guardian’. The study strongly endorses a multipronged strategy of supplementing standard ASV with MAP.

In rural areas with a high incidence of snake bites, compounded by poor access to ASV, oral administration of MAP to N.N bite victims prior to administration of ASV might prove to be a game changer. The compulsive evidence from this study, underscores the merits of using this multipronged strategy in reducing the morbidity, improving survival and thereby decreasing the economic burden on the victim by decreasing hospital-related expenses.

## References

[CR1] Alam MI, Gomes A (1998). Adjuvant effects and antiserum action potentiation by a (herbal) compound 2-hydroxy-4-methoxy benzoic acid isolated from the root extract of the Indian medicinal plant `sarsaparilla’ (Hemidesmus indicus R. Br.). Toxicon.

[CR2] Amos Samkumar R, Premnath D, Raj DP (2019). Strategy for early callus induction and identification of anti-snake venom triterpenoids from plant extracts and suspension culture of Euphorbia hirta L. 3 Biotech.

[CR3] Binder RJ, Karimeddini D, Srivastava PK (2001). Adjuvanticity of α 2 -macroglobulin, an independent ligand for the heat shock protein receptor CD91. J Immunol.

[CR4] Birkenmeier G (1998). Human leptin forms complexes with α2-macroglobulin which are recognized by the α2-macroglobulin receptor/low density lipoprotein receptor-related protein. Eur J Endocrinol.

[CR5] Carvalho BMA (2013). Snake venom PLA2s inhibitors isolated from Brazilian plants: Synthetic and natural molecules. BioMed Res Int.

[CR6] Castro O (1999). Neutralización del efecto hemorrágico inducido por veneno de Bothrops asper (Serpentes: Viperidae) por extractos de plantas tropicales. Rev Biol Trop.

[CR7] Choudhury M (2017). Comparison of proteomic profiles of the venoms of two of the “Big Four” snakes of India, the Indian cobra (*Naja naja*) and the common krait (Bungarus caeruleus), and analyses of their toxins. Toxicon.

[CR8] Coriolano de Oliveira E (2016). Protective effect of the plant extracts of erythroxylum sp. against toxic effects induced by the venom of lachesis muta snake. Molecules.

[CR9] Dar A (2016). Unique medicinal properties of withania somnifera: phytochemical constituents and protein component. Curr Pharm Des.

[CR10] Das P, Srivastav AK (2014). Phytochemical extraction and characterization of the leaves of andrographis paniculata for its anti-bacterial, anti-oxidant, anti-pyretic and anti-diabetic activity. Int J Innov Res Sci Eng Technol.

[CR11] de Silva HJAJA, Ryan NM, de Silva HJAJ (2016). ‘Adverse reactions to snake antivenom, and their prevention and treatment. Br J Clin Pharmacol.

[CR12] Deshpande RP (2013). Adverse drug reaction profile of anti-snake venom in a rural tertiary care teaching hospital. J Young Pharm.

[CR13] Dey YN, Kumari S, Sarada Ota NS (2013). Phytopharmacological review of Andrographis paniculata (Burm. f). Int J Nutr Pharmacol Neurol Dis.

[CR14] Enghild JJ (1989). Proteinase binding and inhibition by the monomeric α-macroglobulin rat α1-inhibitor-3. J Biol Chem.

[CR15] Escalante T (2004). Bothrops asper metalloproteinase BaP1 is inhibited by α 2-macroglobulin and mouse serum and does not induce systemic hemorrhage or coagulopathy. Toxicon.

[CR16] Evans HJ, Guthrie VH (1984). Proteolytic activities of cobra venoms based on inactivation of α2-macroglobulin. Biochim Biophys Acta Protein Struct Mol.

[CR17] Feldman SR, Gonias SL, Pizzo SV (1985). Model of α2-macroglobulin structure and function. Proc Natl Acad Sci USA.

[CR18] Fisker S (2006). Physiology and pathophysiology of growth hormone-binding protein: methodological and clinical aspects. Growth Hormone and IGF Research.

[CR19] Gopi K, Renu K, Jayaraman G (2014). Inhibition of *Naja naja* venom enzymes by the methanolic extract of Leucas aspera and its chemical profile by GC-MS. Toxicol Rep.

[CR20] Gopi K et al (2011) The neutralization effect of methanol extract of Andrographis paniculata on Indian cobra *Naja naja* snake venom. J Pharm Res 4(4): 1010–1012. Available at: http://jprsolutions.info.

[CR21] Ito R, Kuribayashi T (2019). Correlation between synthesis of α2-macroglobulin as acute phase protein and degree of hepatopathy in rats. Lab Anim Res.

[CR22] Kamiguti AS (1994). Ineffectiveness of the inhibition of the main haemorrhagic metalloproteinase from Bothrops jararaca venom by its only plasma inhibitor, α2-macroglobulin. BBA General Subjects.

[CR23] Lonberg-Holm K (1987). Three high molecular weight protease inhibitors of rat plasma. Isolation, characterization, and acute phase changes. J Biol Chem.

[CR24] Lowry OH (1951). Protein measurement with the Folin phenol reagent. J Biol Chem.

[CR25] M SK, M (2013). Andographis paniculata and its bioactive phytochemical constiturents for oxidative damage: a systemic review. Pharmacophore.

[CR26] Meenatchisundaram S (2009). Studies on antivenom activity of Andrographis paniculata and Aristolochia indica plant extracts against Daboia russelli venom by in vivo and in vitro methods. J Sci Technol.

[CR27] Morine N (2008). The occurrence of HR1b in the venom of the snake Okinawa habu (Protobothrops flavoviridis). Biosci Biotechnol Biochem.

[CR28] Nagalekshmi R (2011). Hepatoprotective activity of Andrographis paniculata and Swertia chirayita. Food Chem Toxicol.

[CR29] Nancey S (2008). Serum interleukin-6, soluble interleukin-6 receptor and Crohn’s disease activity. Dig Dis Sci.

[CR30] Naseraldeen N (2021). The role of alpha 2 macroglobulin in igg-aggregation and chronic activation of the complement system in patients with chronic lymphocytic leukemia. Front Immunol.

[CR31] Nayak AG (2020). Can the methanolic extract of Andrographis paniculata be used as a supplement to anti-snake venom to normalize hemostatic parameters: a thromboelastographic study. J Ethnopharmacol.

[CR32] Nayak AG (2020). Anti-snake venom and methanolic extract of Andrographis paniculata: a multipronged strategy to neutralize *Naja naja* venom acetylcholinesterase and hyaluronidase. 3 Biotech.

[CR33] Nayak AG (2021). Evaluation of the merit of the methanolic extract of Andrographis paniculata to supplement anti-snake venom in reversing secondary hemostatic abnormalities induced by *Naja naja* venom. 3 Biotech.

[CR34] Okhuarobo A (2014). Harnessing the medicinal properties of Andrographis paniculata for diseases and beyond: a review of its phytochemistry and pharmacology. Asian Pac J Trop Dis.

[CR35] Paget GE, Barnes JM (1964). Toxicity tests in evaluation of drug activities pharmacometrics. evaluation of drug activities.

[CR36] Peshin YKG, SS, (2012). Do herbal medicines have potential for managing snake bite envenomation? Y. Toxicol Int.

[CR37] Peslova G et al (2009) Hepcidin, the hormone of iron metabolism, is bound specifically to-2-macroglobulin in blood. doi: 10.1182/blood-2009-01-201590. 10.1182/blood-2009-01-201590 19380872

[CR38] Pore SM (2015). A retrospective study of use of polyvalent anti-snake venom and risk factors for mortality from snake bite in a tertiary care setting. Indian J Pharmacol.

[CR39] Premendran SJ (2011). Anti-cobra venom activity of plant Andrographis paniculata and its comparison with polyvalent anti-snake venom. J Nat Sci Biol Med.

[CR40] Rao NR, Bhat PG, Pattabiraman TN (1984). Estimation of serum α2-macroglobulin based on the esterolytic activity of bound α-chymotrypsin. Biochem Med.

[CR41] RehmanAA AH, Khan FH (2013). Alpha-2-macroglobulin: a physiological guardian. J Cell Physiol.

[CR42] Roche M, Pattabiraman TN (1990). Effect of king cobra venom on α2-macroglobulin and proteases in human blood plasma. J Biosci.

[CR43] RS A, S K, P C, S Gnaniah (2014). Isolation, purification and characterization of active compound from Andrographis paniculata L and Phyllanthus amarus L and testing the antivenom activity of the di-herbal extract by invitro and invivo studies. Int Res J Pharm.

[CR44] Saavedra SL (2018). Natural snake venom inhibitors and their pharmaceutical uses: challenges and possibilities. Curr Pharm Des.

[CR45] Sheeja K, Shihab PK, Kuttan G (2006). Antioxidant and anti-inflammatory activities of the plant Andrographis paniculata nees. Immunopharmacol Immunotoxicol.

[CR46] Siddiqui T (2016). Reactive oxygen species and anti-proteinases. Arch Physiol Biochem.

[CR47] Sivakumar A, Manikandan A (2015). Andrographis paniculata leaf extracts as potential *Naja naja* anti-snake venom. World J Pharm Pharmac Sci.

[CR48] Solchaga LA (2012). Safety of recombinant human platelet-derived growth factor-BB in Augment® bone graft. J Tissue Eng.

[CR49] Sujatha S, Jacob RT, Pattabiraman TN (1988). Effect of cobra and viper venoms on α2-macroglobulin activity in human, bovine, and goat sera. Biochem Med Metab Biol.

[CR50] Vandooren J, Itoh Y (2021). Alpha-2-macroglobulin in inflammation, immunity and infections. Front Immunol.

[CR51] Vivas-Ruiz DE (2020). Fibrinogen-clotting enzyme, pictobin, from Bothrops pictus snake venom. Structural and functional characterization. Int J Biol Macromol.

